# Nova Técnica de Preservação do Fluxo na Veia Cefálica durante Implantação de Marca-passo Ipsilateral a Fístula Arteriovenosa

**DOI:** 10.36660/abc.20220926

**Published:** 2023-06-13

**Authors:** Mafalda Carrington, Pedro Silvério António, Natacha Rodrigues, Afonso Nunes-Ferreira, Ana Bernardes, Fausto J. Pinto, João de Sousa, Pedro Marques

**Affiliations:** 1 Hospital do Espírito Santo de Évora Évora Portugal Serviço de Cardiologia, Hospital do Espírito Santo de Évora, Évora – Portugal; 2 Centro Hospitalar Universitário de Lisboa Norte Lisboa Portugal Serviço de Cardiologia, Departamento de Coração e Vasos, Centro Hospitalar Universitário de Lisboa Norte, Lisboa – Portugal; 3 Centro Acadêmico de Medicina de Lisboa Universidade de Lisboa Faculdade de Medicina de Lisboa Lisboa Portugal Centro Acadêmico de Medicina de Lisboa (CAML), Centro Cardiovascular da Universidade de Lisboa, Faculdade de Medicina de Lisboa, Lisboa – Portugal; 4 Departamento de Medicina Centro Hospitalar Universitário de Lisboa Norte Lisboa Portugal Serviço de Nefrologia e Transplante Renal, Departamento de Medicina, Centro Hospitalar Universitário de Lisboa Norte, Lisboa – Portugal

**Keywords:** Marca-Passo Artificial, Fistula Arteriovenosa, Trombose Profunda de Membros Superiores, Diálise Renal

## Introdução

O patrimônio vascular é uma grande preocupação em pacientes com doença renal terminal. Como os pacientes em programas crônicos de hemodiálise experimentam taxas de sobrevivência crescentes, eles frequentemente enfrentam múltiplas falhas de acesso vascular e podem precisar de inserções recorrentes de cateter venoso central e reintervenções de fístulas/enxertos para otimizar seus acessos arteriovenosos. Além disso, insuficiência cardíaca e distúrbios do ritmo cardíaco são comorbidades frequentes nessa população, e o implante de marca-passo ou desfibrilador cardíaco implantável (DCI) é frequentemente indicado.^1^ A prevalência estimada de implante de Dispositivos Eletrônicos Implantáveis Cardiovasculares (DEIC) em pacientes em hemodiálise crônica é de cerca de 10%.^2^ Nosso objetivo foi descrever uma nova técnica de implante de marca-passo, que consistia em preservar a veia cefálica ipsilateral ao fluxo da fístula arteriovenosa, e avaliar os resultados clínicos dessa técnica ao longo de 12 meses.

## Descrição

**Figura 1:**
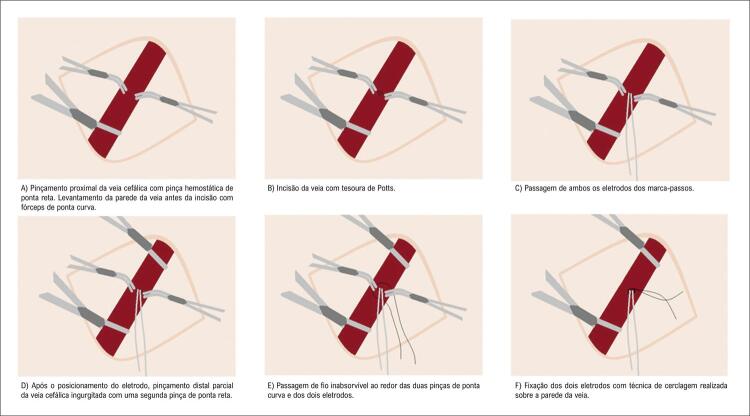
– Representação esquemática das etapas necessárias para realizar a fixação da cerclagem.

**Figura 2:**
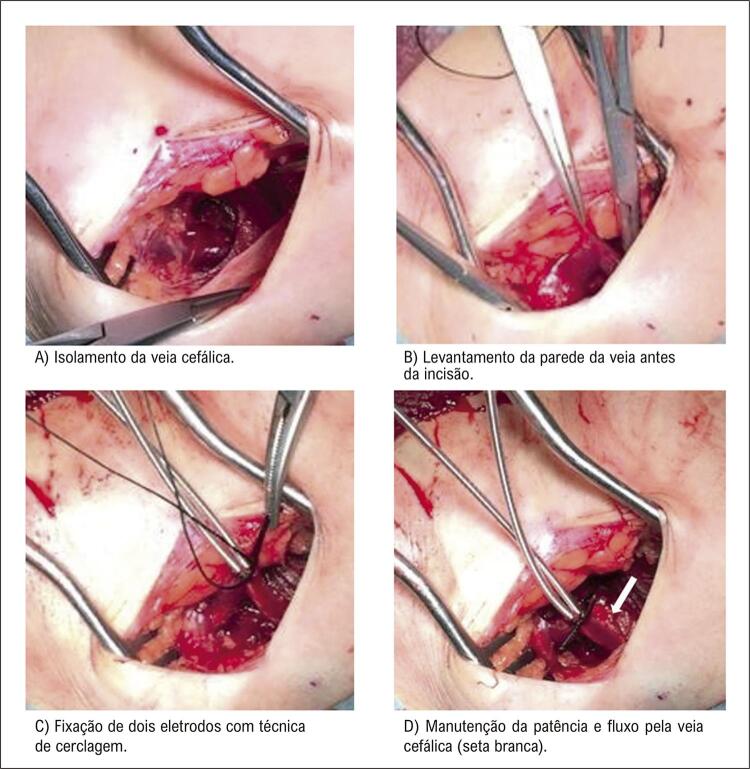
– Fixação do eletrodo do marca-passo com técnica de cerclagem para preservar o fluxo da veia cefálica.

Implantamos um marca-passo endocárdico em cinco pacientes consecutivos em programa crônico de hemodiálise e que apresentavam fístula arteriovenosa radiocefálica esquerda madura (com mais de 2 anos) no antebraço. Quatro receberam dispositivo de dupla câmara com dois eletrodos de fixação ativa 6-French e um recebeu dispositivo de câmara única com o mesmo tipo de eletrodo. Em vez de interromper o fluxo da veia cefálica para fixação do eletrodo, isso foi feito com a técnica de sutura de cerclagem, permitindo assim a patência e a manutenção do fluxo pelo vaso em todos os cinco pacientes ( Figura 1 ). Após desbridamento tecidual e isolamento da veia cefálica, a veia foi clampeada proximalmente com pinça hemostática de ponta reta. A seguir, duas pinças de ponta curva foram usadas para descolar a parede da veia e uma tesoura de Potts foi usada para cortá-la e ter acesso ao lúmen do vaso. Ambos os eletrodos foram inseridos na veia e posicionados no átrio e ventrículo direitos. Por fim, um fio inabsorvível foi colocado ao redor da pinça de ponta curva e os dois eletrodos e a cerclagem foram apertados sobre a parede da veia para fixação dos eletrodos ( Figura 2 ).

Durante um período de acompanhamento de 12 meses, a avaliação clínica e as complicações foram observadas. A avaliação do fluxo da fístula arteriovenosa, bolso do marca-passo, posicionamento do eletrodo e desempenho foram registrados prospectivamente.

Os programas de hemodiálise não foram interrompidos e todos os tratamentos foram realizados com a fístula arteriovenosa original. Todos os pacientes foram submetidos a técnicas mensais de vigilância do fluxo da fístula arteriovenosa (medidas de Qa por ultrassonografia Doppler^3^ e avaliação clínica semanal, e nenhuma alteração foi registrada durante o período de acompanhamento ( Tabela 1 ). Além disso, não foram observadas complicações locais relacionadas à bolsa do marca-passo ou ao posicionamento do eletrodo na radiografia. Finalmente, nenhum dos pacientes apresentou evidência de disfunção do eletrodo no final do primeiro mês ou nas visitas de acompanhamento do marca-passo no primeiro ano.

**Tabela 1:** – Características do paciente e acompanhamento

Gênero	Idade	Indicação de MP	Dispositivo de câmara única ou dupla	Tempo desde a 1ª canulação da fístula arteriovenosa (no implante do MP)	Qa pré-implantação (ml/min)	Variação máxima de Qa pós-implantação (%)	Duração do acompanhamento
Masc.	68	Bloqueio AV completo	Dupla	6 anos	1132	-5%	12 meses
Fem.	52	Bloqueio AV completo	Dupla	2 anos	988	+9%	12 meses
Masc.	73	Bloqueio AV 2:1	Dupla	5 anos	781	-7%	12 meses
Masc.	71	Bloqueio AV completo	Dupla	>10 anos	1780	-3%	12 meses
Masc.	80	Bradi-AF	Única	>10 anos	699	-11%	12 meses

FA: fibrilação atrial; AV: auriculoventricular; MP: marca-passo. As medições de Qa relatadas representam a média de três valores obtidos para cada paciente

## Discussão

Embora a trombose venosa profunda crônica relacionada ao marca-passo seja uma complicação bem conhecida (descrita em 21-45% dos pacientes), a maioria permanece assintomática devido ao desenvolvimento de circulação colateral venosa adequada.^4 - 7^ No entanto, a trombose da veia subclávia pode ter sérias implicações quando o paciente apresenta uma fístula arteriovenosa ipsilateral concomitante, embora não haja evidências suficientes para recomendar a implantação do dispositivo contralateral a uma fístula arteriovenosa.^3^ Pequenos estudos observacionais demonstraram que a construção de fístula arteriovenosa em pacientes com DCEI pode aumentar a falha da fístula no mesmo lado,^8^ e que há maior incidência de estenose venosa central em pacientes com DCEI ipsilateral e fístula arteriovenosa, em comparação com pacientes com estratégia contralateral.^9^ Em uma coorte retrospectiva,^2^ houve taxas mais altas de intervenção na veia central nos casos ipsilaterais, ainda que a necessidade de intervenção nos acessos arteriovenosos para hemodiálise fosse semelhante em ambos os grupos. Nesse estudo, os casos ipsilaterais corresponderam mais frequentemente a pacientes em que as fístulas arteriovenosas foram construídas após implante de DCEI (81%) e os casos contralaterais a pacientes com fístulas arteriovenosas anteriores ao implante de DCEI (56%), sugerindo assim que a maturidade da fístula arteriovenosa pode desempenhar um papel importante na prevenção da trombose venosa profunda e na necessidade de intervenção. De fato, os enxertos maduros podem estar associados ao aumento do fluxo e diâmetro da veia cefálica, contribuindo assim para a patência do acesso. Nossa hipótese é que a avaliação com Doppler da veia cefálica pode ser útil para recomendar a implementação desta técnica inovadora de cerclagem.

Por outro lado, embora as complicações infecciosas sejam relativamente pouco frequentes na população geral com dispositivos, os pacientes com doença renal em estágio terminal têm um risco 9 vezes maior de infecção por DEIC.^10^

Alguns autores defendem que alternativas como eletrodos epicárdicos, DCI subcutâneo,^11^ ou marca-passos sem eletrodos^12^ devem ser usadas em pacientes com doença renal crônica.^13^ No entanto, tais dispositivos sem eletrodos intracavitários são menos disponíveis e mais caros, e não permitem tanto a detecção do átrio quanto a estimulação. Embora os marca-passos sem eletrodos pareçam ter um perfil de segurança aceitável e um baixo risco de infecção,^12^ faltam evidências de seu benefício e segurança em pacientes em hemodiálise altamente comórbidos, pois esses pacientes foram sub-representados em ensaios clínicos. Além disso, embora os eletrodos do marca-passo epicárdico e do DCI subcutâneo não sejam intravasculares e, portanto, não sejam suscetíveis à colonização bacteriana e endocardite, os geradores de pulso também podem estar sujeitos a infecção de bolsa. A maioria das complicações infecciosas em pacientes com DCEI está relacionada à infecção de bolsa, conforme demonstrado em uma revisão retrospectiva de todos os pacientes com infecções de dispositivos cardíacos internados na Mayo Clinic, onde a incidência de infecção de bolsa (com ou sem bacteremia) esteve presente em quase três quartos.^14^

Finalmente, a trombose venosa profunda do lado da fístula arteriovenosa poderia ser minimizada pela punção da veia axilar maior ou implante no lado contralateral. No entanto, nenhuma das opções reduziria o risco de infecção principalmente associado ao acesso vascular repetido durante a diálise.^15^ Outra desvantagem da última estratégia é que o DCI no lado direito frequentemente resulta em limiares de desfibrilação mais altos, exigindo assim testes de limiar de desfibrilação.^16^

## Conclusão

Com esta série de casos, pretendemos demonstrar que, em pacientes com fístulas maduras, o implante de marca-passo com técnica que preserva o fluxo da veia cefálica pode ser seguro e inofensivo para uma fístula arteriovenosa ipsilateral. Essa estratégia simples parece permitir a preservação do patrimônio vascular contralateral sem comprometer o programa de diálise estabelecido, tornando-se uma possível alternativa aos dispositivos sem eletrodos.
