# Supplementary material for: Nova Técnica de Preservação do Fluxo na Veia Cefálica durante Implantação de Marca-passo Ipsilateral a Fístula Arteriovenosa
Source: Arq Bras Cardiol. 2023 Jun 13;120(5):e20220926. [Article in Portuguese] doi: 10.36660/abc.20220926 (PMC12080601; doi:10.36660/abc.20220926)
Supplement: Translation

# Translation: New Technique for Preserving Cephalic Vein Flow in Pacemaker Implantation Ipsilateral to Arteriovenous Fistula

**Authors:** Mafalda Carrington, Pedro Silvério António, Natacha Rodrigues, Afonso Nunes-Ferreira, Ana Bernardes, Fausto J. Pinto, João de Sousa, Pedro Marques

**Affiliations:** 1 Portugal Serviço de Cardiologia, Hospital do Espírito Santo de Évora, Évora – Portugal; 2 Portugal Serviço de Cardiologia, Departamento de Coração e Vasos, Centro Hospitalar Universitário de Lisboa Norte, Lisboa – Portugal; 3 Portugal Centro Acadêmico de Medicina de Lisboa (CAML), Centro Cardiovascular da Universidade de Lisboa, Faculdade de Medicina de Lisboa, Lisboa – Portugal; 4 Portugal Serviço de Nefrologia e Transplante Renal, Departamento de Medicina, Centro Hospitalar Universitário de Lisboa Norte, Lisboa – Portugal

**Keywords:** Pacemaker, Artificial, Arteriovenous Fistula, Upper Extremity Deep Vein Thrombosis, Renal Dialysis

## Introduction

Vascular patrimony is a major concern in patients with end-stage renal disease. As patients on chronic hemodialysis programs experience increasing survival rates, they frequently face multiple vascular access failures and may need recurrent central venous catheter insertions and fistula/grafts re-interventions to optimize their arteriovenous accesses. In addition, heart failure and heart rhythm disorders are frequent comorbidities in this population, and pacemaker or implantable cardiac defibrillator (ICD) implantation are frequently indicated.^1^ The estimated prevalence of Cardiovascular Electronic Implantable Devices (CIED) implantation in patients on chronic hemodialysis is about 10%.^2^ We aimed to describe a new technique for pacemaker implantation, which consisted in preserving cephalic vein ipsilateral to arteriovenous fistula flow, and in evaluating clinical outcomes of this technique over 12 months.

## Description

We implanted an endocardial pacemaker in five consecutive patients in a chronic hemodialysis program and that had a mature (with more than 2 years) left radiocephalic arteriovenous fistula in the forearm. Four received a double chamber device with two 6-French pacings active fixation leads, and one received a single chamber device with the same lead type. Instead of interrupting the cephalic vein flow for lead fixation, this was accomplished with a cerclage suture technique, thus allowing patency and flow maintenance through the vessel in all five patients ( Figure 1 ). After tissue debridement and cephalic vein isolation, the vein was proximally clamped with straight-tip hemostatic forceps. Subsequently, two curved-tip forceps were used to detach the vein wall, and a Potts scissor was used to cut it and gain access to the vessel’s lumen. Both leads were inserted into the vein and positioned in the right atrium and ventricle. Finally, a non-absorbable thread was placed around the curved-tip forceps, and the two leads and the cerclage were tightened over the vein wall to fixate the electrodes ( Figure 2 ).

**Figure 1:** – Schematic representation of the steps necessary to perform the cerclage fixation.

**Figure 2:** – Pacemaker lead fixation with a cerclage technique to preserve cephalic vein flow.

Over a 12-month follow-up period, clinical evaluation and complications were observed. Arteriovenous fistula flow assessment, pacemaker pocket, lead positioning, and performance were prospectively registered.

Hemodialysis programs were not interrupted, and all treatments were performed using the original arteriovenous fistula. All patients underwent monthly surveillance techniques of arteriovenous fistula flow (Qa measurements by Doppler ultrasound^3^ and weekly clinical evaluation, and no changes were recorded during the follow-up period ( Table 1 ). Moreover, no local complications concerning pacemaker pocket or lead positioning on x-ray were observed. Finally, none of the patients had evidence of lead dysfunction at the end of the first month or the first-year pacemaker follow-up visits.

**Table 1:** – Patient characteristics and follow-up

Genre	Age	PM indication	Single or double-chamber device	Time since 1^**st**^ arteriovenous fistula cannulation (at PM implantation)	Pre-implantation Qa (ml/min)	Maximum post-implantation Qa variation (%)	Follow-up duration
Male	68	Complete AV block	Double	6 years	1132	-5%	12 months
Female	52	Complete AV block	Double	2 years	988	+9%	12 months
Male	73	2:1 AV block	Double	5 years	781	-7%	12 months
Male	71	Complete AV block	Double	>10 years	1780	-3%	12 months
Male	80	Bradi-AF	Single	>10 years	699	-11%	12 months

AF: atrial fibrillation; AV: auriculoventricular; PM: pacemaker. Reported Qa measurements represent the mean of three values obtained for each patient.

## Discussion

Although pacemaker lead-related chronic deep venous thrombosis is a well-known complication (described in 21-45% of the patients), most remain asymptomatic due to the development of adequate venous collateral circulation.^4 - 7^ Yet, subclavian vein thrombosis may have serious implications when the patient has a concomitant ipsilateral arteriovenous fistula, although there is not enough evidence to recommend device implantation contralateral to an arteriovenous fistula.^3^ Small observational studies have demonstrated that arteriovenous fistula construction in patients with CIED may increase primary arteriovenous fistula failure on the same side,^8^ and that there is a higher incidence of central venous stenosis in patients with ipsilateral CIED and arteriovenous fistula, comparing to patients with contralateral strategy.^9^ In a retrospective cohort,^2^ there were higher rates of central vein intervention in ipsilateral cases, even if the need for intervention in arteriovenous hemodialysis accesses were similar in both groups. In that study, ipsilateral cases corresponded more frequently to patients in which arteriovenous fistulas were constructed after CIED implantation (81%) and contralateral cases to patients with arteriovenous fistulas prior to CIED implantation (56%), thus suggesting that the maturity of the arteriovenous fistula may play an important role in the prevention of deep venous thrombosis and need for intervention. In fact, mature grafts may be associated with increased cephalic vein flow and diameter, thus contributing to access patency. We hypothesize that cephalic vein Doppler ultrasound evaluation could be useful to recommend implementing this innovative cerclage technique.

On the other hand, although infectious complications are relatively infrequent in the overall population with devices, patients with end-stage renal disease have a 9-fold higher risk of CIED infection.^10^

Some authors argue that alternatives such as epicardial leads, subcutaneous ICD^11^ or leadless pacemakers^12^ should be used in patients with chronic kidney disease.^13^ However, such devices without intracavitary leads are less available and more expensive, and they do not allow both atrium sensing and pacing. Although leadless pacemakers appear to have an acceptable safety profile and a low risk of infection,^12^ evidence of their benefit and safety in highly-comorbid hemodialysis patients is lacking, as these patients were underrepresented in clinical trials. In addition, although epicardial pacemaker and subcutaneous ICD leads are not intravascular and thus not susceptible to bacterial colonization and endocarditis, pulse generators may also be subject to pocket infection. The majority of infectious complications in patients with CIED are related to pocket infection, as demonstrated in a retrospective review of all patients with cardiac device infections admitted to the Mayo Clinic, where an incidence of pocket infection (with or without bacteriemia) was present in almost three quarters.^14^

Finally, deep venous thrombosis on the side of the arteriovenous fistula could be minimized by puncture of the larger axillary vein or implantation on the contralateral side. However, none of the options would lower the infection risk mostly associated with repeated vascular access during dialysis.^15^ Another disadvantage of the latter strategy is that ICD on the right side frequently results in higher defibrillation thresholds, thus requiring defibrillation threshold testing.^16^

## Conclusion

With this case series, we intended to demonstrate that, in patients with mature fistulas, pacemaker implantation with a technique that preserves the cephalic vein flow may be safe and unharmful to an ipsilateral arteriovenous fistula. This simple strategy seems to allow the preservation of the contralateral vascular patrimony without compromising the established dialysis program, thus becoming a possible alternative to leadless devices.
